# Sustainable efficacy of nusinersen in children with later-onset spinal muscular atrophy: a 3-year observational study

**DOI:** 10.1007/s12519-025-00938-y

**Published:** 2025-07-29

**Authors:** Yi-Jie Feng, Yi-Cheng Yu, Jing-Li Zhao, Lu Xu, Cong-Ying Zhao, Yi Hua, Guo-Xia Sheng, Ting-Ting Chen, Yi Zhang, Lin-Lin Wei, Feng Gao, Shan-Shan Mao

**Affiliations:** 1 Department of Neurology, Children’s Hospital, Zhejiang University School of Medicine, National Clinical Research Center for Child Health, Hangzhou 310052, China; 2 Department of Nephrology, Children’s Hospital, Zhejiang University School of Medicine, National Clinical Research Center for Child Health, Hangzhou 310052, China; 3 Department of Developmental Behavior, Children’s Hospital, Zhejiang University School of Medicine, National Clinical Research Center for Child Health, Hangzhou 310052, China

Spinal muscular atrophy (SMA) is a fatal and disabling hereditary disease characterized by progressive motor dysfunction caused by survival motor neuron 1 (*SMN1*) gene deletion or mutation [1]. Childhood-onset SMA is typically classified into types 1–3, with types 2 and 3 referred to as later-onset SMA [2]. Nusinersen, the first drug approved as a disease-modifying therapy (DMT) for SMA [3], is an antisense oligonucleotide that promotes increased production of the full-length SMN protein by modifying pre-messenger RNA splicing of the *SMN2* gene to improve the function of motor neurons and alleviate disease symptoms [4]. Previous real-world studies have reported the efficacy of nusinersen in patients with SMA [5] and explored the value of various factors, such as the disease duration at DMT initiation and the baseline score of motor function, in evaluating treatment efficacy and predicting changes in motor function [6, 7]. However, as nusinersen has been covered by national medical insurance in China since 2022 and then commonly used in clinical practice, few longer-term follow-up studies on Asian patient populations are available on the evolution of motor function, the sustainability of efficacy, and the related predictive indicators in Chinese patients with SMA receiving nusinersen treatment.

This study quantitatively analyzed the long-term efficacy of nusinersen treatment on motor function in Chinese patients with later-onset SMA who were treated with nusinersen during a 3-year follow-up period. The potential predictive effects of disease duration and the baseline score on treatment efficacy were further explored.

Data from 50 children with SMA types 2 and 3 who were treated with nusinersen and followed up from December 2020 to October 2024 were analyzed. This study was approved by the Institutions’ Ethical Committee of our hospital. Informed consent was obtained from the patients or their legal guardians. The inclusion criteria were as follows: (1) children aged < 18 years; (2) those genetically diagnosed with 5qSMA; and (3) those with a spinal Cobb angle of no more than 50° and without severe contractures or a history of spinal surgery [8]. Children with a history of DMT before, concomitant disease or other drug treatment, which may influence the results of efficacy, and those who could not complete the scale evaluation, for example, patients with severe scoliosis with a Cobb angle of more than 50°, a history of spinal surgery or severe contractures that affect the movement of limbs and joints, were excluded [9].

Nusinersen was intrathecally injected through lumbar puncture at a dosage of 12 mg on days 0, 14, 28, and 63, followed by maintenance doses every 4 months [10]. The patients were monitored for potential adverse events after the injections. The motor function evaluations were conducted at baseline and at 8 follow-up time points: 6 (dose 5, abbreviated D5), 10 (D6), 14 (D7), 18 (D8), 22 (D9), 26 (D10), 30 (D11) and 34 (D12) months. The Hammersmith Functional Motor Scale Expanded (HFMSE) scale was used to evaluate the motor function of patients, and the Revised Upper Limb Module (RULM) scale was further applied to those who could sit independently for a while [9]. Higher scores indicate better motor function. A positive response was defined as an improvement of ≥ 3.0 points in the HFMSE or ≥ 2.0 points in the RULM [11]. Additionally, patients with SMA type 3 who could walk independently for a while and cooperate with the evaluation also completed the 6-minute walking test (6MWT) during the follow-up. Disease duration is referred to as the time window from the disease onset of clinical symptoms in SMA patients to the initiation of disease-modifying treatment. The statistical analyses were performed via R statistical software (version 4.3.0) and SPSS software (version 26.0). Independent sample *t* tests or rank sum tests were used for intergroup comparisons, and paired sample *t* tests or rank sum tests were used for intragroup comparisons during follow-up. A linear mixed model (LMM) was employed to estimate the population-average change in scale scores from baseline to the last follow-up. A *P* < 0.05 was considered significant.

The baseline characteristics of the 50 patients are presented in Table 1. The age of onset differed between patients with SMA types 2 and 3 (*P* < 0.001), while no significant difference was found in the age of initial treatment or disease duration (*P* = 0.745 and 0.771, respectively). When the nutritional status of these patients under treatment was monitored from baseline to the last follow-up, no significant difference was found (all *P* > 0.05). The patients’ baseline motor function scale scores are listed in Table 2.

**Table 1:** Baseline characteristics of patients with SMA

Variables	All (*N* = 50)	Type 2 (*n* = 32)	Type 3 (*n* = 18)
Age of onset (y)	1.00 (0.73–1.50)	0.81 (0.64–1.02)	1.50 (1.27–2.57)
Age of initial treatment (y)	5.84 (3.42–9.11)	4.60 (2.53–7.65)	7.81 (3.79–10.67)
*SMN2* copy number			
2	4 (8.0)	4 (12.5)	0 (0.0)
3	46 (92.0)	28 (87.5)	18 (100.0)
Disease duration (y)	4.63 (2.39–7.80)	4.01 (1.89–7.03)	6.31 (2.60–8.58)
Sex			
Male	22 (44.0)	16 (50.0)	6 (33.3)
Female	28 (56.0)	16 (50.0)	12 (66.7)
Mobility milestone			
Sitter	32 (64.0)	32 (64.0)	0 (0.0)
Walker	18 (36.0)	0 (0.0)	18 (36.0)
BMI (kg/m^2^)	16.01(13.97–18.25)	15.61 (13.45–16.73)	16.97 (14.99–18.60)
Nutritional status			
Malnutrition	11 (22.0)	9 (28.1)	2 (11.1)
Normal	31 (52.0)	19 (59.4)	12 (66.7)
Overnutrition	8 (16.0)	4 (12.5)	4 (22.2)
Rehabilitation			
No	22 (43.1)	18 (51.4)	4 (25.0)
Yes	29 (56.9)	17 (48.6)	12 (75.0)

The data are expressed as *n* (%) or median (range). Disease duration is referred to as the time window from the disease onset of clinical symptoms of SMA patients to the initiation of disease-modifying treatment. Mobility milestone is referred to as the highest functional capacity of SMA patients ever achieved. Malnutrition was defined as a height-for-age *Z*-score or weight-for-height Z-score (WHZ) or BMI-for-age Z-score (BAZ) ≤  − 2, while over-nutrition was defined as WHZ or BAZ ≥ 2. Rehabilitation was considered present if it was initiated before the DMT initiation, and was conducted on the motor function frequently at least 3 times per week according to the recommended standard of care in SMA. Continuous variables with normal distribution were expressed as mean ± standard deviation and those with abnormal distribution were expressed as median (Q1-Q3), whereas categorical variables were expressed as count (percentage). *SMA* spinal muscular atrophy, *SMN* survival motor neuron, *BMI* body mass index, *DMT* disease-modifying therapy

**Table 2:** Changes in HFMSE and RULM scores from baseline to different follow-up time point

Variables		HFMSE			RULM						
		All (*n* = 50)	SMA type 2 (*n* = 32)	SMA type 3 (*n* = 18)	All (*n* = 32)				SMA type 2 (*n* = 14)		SMA type 3 (*n* = 18)
Baseline score^a^		18.5 (7.5–36.5)	11.0 (5.0–18.8)	39.2 ± 11.8	21.7 ± 8.3				14.5 ± 4.5		27.2 ± 6.0
6 mon											
Score		20.5 (10.0–38.3)	13.0 (5.3–20.8)	42.1 ± 11.7	23.1 ± 8.6				15.4 ± 4.9		29.0 ± 5.6
Change value^b^		2.5 (1.4–3.6)^‡^	2.3 (1.0–3.7)^‡^	2.8 (1.0–4.7)^‡^	1.4 (0.5–2.3)^‡^				0.9 (− 0.5, 2.4)		1.8 (0.6–3.0)^†^
10 mon											
Score		22.0 (11.5–40.0)	15.0 (6.3–21.8)	43.1 ± 11.7	24.1 ± 8.2				16.6 ± 4.2		29.8 ± 5.4
Change value		3.6 (2.5–4.6)^‡^	3.4 (2.1–4.8)^‡^	3.8 (2.0–5.7)^‡^	2.4 (1.5–3.3)^‡^				2.1 (0.7–3.6)^†^		2.6 (1.4–3.8)^‡^
14 mon											
Score		23.0 (12.8–42.5)	15.0 (7.3–23.0)	44.5 ± 11.4	25.2 ± 7.8				18.0 ± 4.0		30.8 ± 4.9
Change value		4.7 (3.8–6.0)^‡^	4.6 (3.3–6.0)^‡^	5.3 (3.4–7.1)^‡^	3.5 (2.6–4.5)^‡^				3.5 (2.1–4.9)^‡^		3.6 (2.4–4.8)^‡^
18 mon											
Score		24.0 (13.8–44.3)	16.5 (7.3–24.0)	45.6 ± 11.5	25.7 ± 7.6				18.7 ± 4.1		31.1 ± 4.7
Change value		6.0 (4.9–7.1)^‡^	5.8 (4.4–7.1)^‡^	6.4 (4.6–8.2)^‡^	4.0 (3.1–4.9)^‡^				4.2 (2.8–5.7)^‡^		3.8 (2.6–5.0)^‡^
22 mon											
Score		25.0 (14.0–47.3)	18.0 (10.0–25.8)	47.2 ± 11.2	26.5 ± 7.5				19.6 ± 4.0		31.8 ± 4.8
Change value		7.4 (6.3–8.5)^‡^	7.1 (5.7–8.4)^‡^	8.0 (6.2–9.8)^‡^	4.8 (3.9–5.8)^‡^				5.1 (3.7–6.6)^‡^		4.6 (3.4–5.8)^‡^
26 mon											
Score		25.5 (14.0–48.0)	18.0 (12.0–26.8)	47.9 ± 11.2	27.5 ± 7.2				20.9 ± 3.7		32.6 ± 4.6
Change value		8.1 (7.0–9.2)^‡^	7.8 (6.4–9.1)^‡^	8.7 (6.9–10.6)^‡^	5.8 (4.9–6.7)^‡^				6.4 (5.0–7.9)^‡^		5.3 (4.1–6.5)^‡^
30 mon											
Score		26.5 (15.0–49.3)	19.0 (13.0–27.8)	48.8 ± 11.0	28.3 ± 7.2				21.7 ± 3.7		33.5 ± 4.4
Change value		9.1 (7.8–10.4)^‡^	8.9 (7.2–10.5)^‡^	9.6 (7.2–11.9)^‡^	6.7 (5.6–7.8)^‡^				7.2 (5.9–8.6)^‡^		6.3 (4.5–8.0)^‡^
34 mon											
Score		28.0 (15.8–50.3)	20.0 (14.3–29.0)	49.6 ± 11.0	29.1 ± 7.1				22.6 ± 3.7		34.1 ± 4.4
Change value		10.1 (8.8–11.4)^‡^	9.9 (8.3–11.5)^‡^	10.4 (7.8–13.0)^‡^	7.4 (6.2–8.6)^‡^				8.1 (6.5–9.6)^‡^		6.9 (5.0–8.8)^‡^

Continuous variables with normal distribution were expressed as mean ± standard deviation and those with abnormal distribution were expressed as median (Q1-Q3). Paired sample *t*-test or rank sum test was used for intragroup comparison during the follow-up. *SMA* spinal muscular atrophy, *HFMSE* Hammersmith Functional Motor Scale Expanded (maximum score of 66 points), *RULM* Revised Upper Limb Module (maximum score of 37 points). ^a^Data are expressed as median (Q1-Q3) or mean (± standard deviation); ^b^data are mean change versus baseline (95% confidence interval). ^*^P < 0.05, ^†^*P* < 0.01, ^‡^*P* < 0.001

The HFMSE and RULM scores collected from 8 visits of each patient exhibited an overall upward trend or remained stable during the 3-year follow-up period (Supplementary Fig. 1). Furthermore, the HFMSE scores significantly increased at each follow-up time point compared with those at baseline (Table 2). In addition, at almost every follow-up time point, both patients with SMA type 2 and type 3 had significant increases in HFMSE and RULM scores compared with those at baseline. Four children with SMA type 3 completed the 6MWT with baseline walking distances of 360 m, 82.4 m, 378.5 m and 197.3 m, and the results at the last follow-up increased to 495 m, 135.6 m, 474.6 m and 257.8 m, respectively. Compared with those at baseline, the walking distances of all four children continuously improved to varying degrees.

Overall, the proportion of positive responders to the HFMSE scores increased from 34.0% (17/50) at D5 to 98.0% (49/50) at D11, whereas that to the RULM scores increased from 40.6% (13/32) to 93.8% (30/32) (Supplementary Fig. 2). The responders to the scale scores remained stable from D10 to D12. To supplement these findings, four of the patients with SMA type 2 have achieved motor milestone breakthrough (gaining walking function) since the 10th injection was completed.

According to the LMM analysis (Table 3), patients with a shorter disease duration presented significantly higher HFMSE scores than those with a longer disease duration did, and the differences between the two groups became increasingly greater within 34 months, with average monthly changes of 0.180 [95% confidence interval (CI) = − 0.239 to − 0.122] and 0.223 points (95% CI = − 0.300 to − 0.146) for SMA types 2 and 3, respectively. However, no significant differences in the HFMSE scores were observed between the patients with higher and lower baseline scores for SMA types 2 and 3 (all *P* > 0.05).

**Table 3:** Associations between disease duration or baseline score and HFMSE score derived from a linear mixed model

	Disease duration^a^				Baseline score^b^			
Outcome	Intercept	Time	Disease duration	Time × disease duration	Intercept	Time	Baseline score	Time × baseline score
Overall patients								
* β*	9.952	0.589	1.845	− 0.240	9.156	0.286	− 2.646	0.054
SE	2.931	0.044	1.888	0.062	2.164	0.046	2.332	0.644
95% CI	4.036, 15.868	0.809, 0.981	− 5.674, 1.956	− 0.462, − 0.218	4.791, 13.520	0.996, 1.176	− 4.304, 1.013	−0.181, 0.073
* P*	0.002	< 0.001	0.334	< 0.001	< 0.001	< 0.001	0.221	0.403
SMA type 2								
* β*	9.209	0.378	2.411	− 0.180	10.611	0.272	− 3.207	0.107
SE	3.565	0.020	1.909	0.030	3.366	0.018	2.091	0.036
95% CI	1.821, 16.597	0.339, 0.416	− 1.528, 6.351	− 0.239, − 0.122	3.635, 17.588	0.236, 0.308	− 7.515, 1.100	0.036, 0.178
* P*	0.017	< 0.001	0.219	< 0.001	0.005	< 0.001	0.138	0.303
SMA type 3								
* β*	4.465	0.466	1.083	− 0.223	8.733	0.225	− 2.727	0.111
SE	2.131	0.090	2.344	0.039	4.165	0.090	4.510	0.093
95% CI	− 0.298, 9.228	0.406, 0.527	− 4.139, 6.304	− 0.300, − 0.146	− 0.444, 17.909	0.046, 0.404	− 12.688, 7.235	− 0.074, 0.295
* P*	0.063	< 0.001	0.654	< 0.001	0.060	0.014	0.558	0.238

The fixed effect of the model was the time since the first injection. The random intercept of each patient’s baseline score was added as a random effect. Patients were grouped on the basis of the baseline HFMSE score (≥ median score vs. < median score) or disease duration (≥ median duration vs. < median duration), and the LMM was also used to analyze the correlations of disease duration and baseline score with the changes in HFMSE scores, as well as their interactions with time, adjusting for confounding factors, including sex, mobility status, nutritional status, age of onset and initial treatment, and rehabilitation status. Intercept, the mean HFMSE score at baseline in the group of disease duration shorter than median or in the group of baseline scores lower than median; time, the mean changes in HFMSE scores over time (monthly) in the group of disease duration shorter than median or in the group of baseline scores lower than median; disease duration, the difference in HFMSE scores at baseline between the groups of disease duration shorter than median and disease duration longer than median; baseline score, the difference in HFMSE scores at baseline between the groups of baseline score lower than median and baseline score higher than median; time × disease duration, the mean changes in HFMSE scores monthly in the group of disease duration longer than median compared with the group of disease duration shorter than median; time × baseline score, the mean changes in HFMSE scores monthly in the group of baseline score higher than median compared with the group of baseline score lower than median. *HFMSE* Hammersmith functional motor scale expanded, *LMM* linear mixed model, *SMA* spinal muscular atrophy, *SE* standard error, *CI* confidence interval. ^a^The model was adjusted for sex, mobility status, baseline HFMSE score, nutritional status, age of onset, age of initial treatment, and rehabilitation status; ^b^the model was adjusted for sex, mobility status, disease duration, nutritional status, age of onset, age of initial treatment, and rehabilitation status

In total, 600 injections were performed during the 3-year follow-up, with a total adverse event rate of 26.0% (13/50) reported by patients. Back pain (10.0%) and headache (8.0%) were the most common adverse events (Supplementary Table 1); no severe adverse events occurred. No one discontinued treatment owing to adverse events.

To date, there have been no other studies on the efficacy of nusinersen in children with SMA or its predictive indicators for longer-term outcomes in the Chinese population. This 3-year follow-up study reported a significant and continued improvement in motor scores at each follow-up time point in SMA patients receiving nusinersen. Furthermore, longitudinal analyses quantitatively revealed that a shorter disease duration was associated with greater improvements in motor function following treatment, suggesting that early diagnosis and treatment are important for treatment efficacy.

In China, nusinersen was the first drug applied as a DMT for SMA. Studies have shown that the motor function of patients with later-onset SMA exhibits a progressive nonlinear decline during their natural history [12]; however, patients receiving nusinersen experience notable improvements in motor function [10, 13, 14]. In this study, the overall nusinersen-treated patients had significant improvements in HFMSE scores, suggesting that nusinersen can effectively ameliorate the motor function of SMA patients, which is consistent with the findings of previous studies [15]. The follow-up outcomes of the four patients with SMA type 2 who acquired walking ability further prove that continuous DMT can help achieve motor function breakthrough and improve patient prognosis. Furthermore, the RULM scores also significantly increased, supporting previous findings that long-term nusinersen treatment can effectively improve upper limb function [16–18]. Moreover, individual motor scores showed an overall upward trend or remained stable, implying a continued therapeutic effect. Although several patients with SMA type 3 showed no significant changes in RULM scores during the follow-up, the results suggested that their upper limb function remained at a stable high level under nusinersen treatment. Compared with the gradual motor function decline in patients during their natural history [12], these findings also suggest that there is a sustainable benefit for these patients [7].

Although previous studies reported response rates of 24.6%–49.0% for HFMSE [16, 18] and 17.0%–37.0% for RULM [16, 17], our study revealed rates of 98% and 93.8% for HFMSE and RULM, respectively, at the 26-month visit and subsequent visits. This variation may be attributed to differences in assessment time points and individual characteristics, including disease subtypes, age, and baseline scores. Although patients with SMA types 2 and 3 benefit from nusinersen therapy, differences exist in the evolution of their motor function over time, such as the distinct points of significant improvement in motor scores and response rates at each time point, suggesting potential subtype-specific variations in nusinersen efficacy, as reported previously [6, 15].

The LMM analysis further revealed that a negative association between disease duration and improvement in motor function following treatment was observed among these patients, showing that children with shorter disease durations tend to achieve better improvements in motor function following treatment, similar to previous findings [6, 7], highlighting the significance of early diagnosis and intervention for SMA [10]. However, apart from previous findings, no associations were observed between baseline scores and changes in motor function [16, 17]. Notably, previous studies included adult patients, potentially leading to differences in the results, also indicating that early DMT in childhood could achieve a better response than starting in adulthood.

With respect to drug safety, the most commonly reported adverse events are related to lumbar punctures, including back pain and headache, with a frequency similar to that caused by routine lumbar punctures [14]. Our findings further proved the safety of nusinersen therapy on the basis of previous studies [19].

The limitations of this study include its retrospective cohort design, single-center design, and limited sample size. In conclusion, nusinersen treatment is not only effective but also sustainable in improving and maintaining the motor function of Chinese children with later-onset SMA. To achieve better efficacy in clinical practice, early diagnosis and treatment of SMA are crucial.

## Supplementary Information

Below is the link to the electronic supplementary material.

Supplementary file 1 (PDF 429 KB)

## Data Availability

The data sets generated during and/or analyzed during the current study are available from the corresponding author upon reasonable request.
