# Supplementary material for: Sustainable efficacy of nusinersen in children with later-onset spinal muscular atrophy: a 3-year observational study
Source: World J Pediatr. 2025 Jul 29;21(8):846–51. doi: 10.1007/s12519-025-00938-y (PMC12380860; doi:10.1007/s12519-025-00938-y)
Supplement: Supplementary file 1 — (PDF 429 KB) [file 12519_2025_938_MOESM1_ESM.pdf]

# Supplementary materials

**Supplementary Table** 1. Adverse events reported by patients with SMA under nusinersen treatment during 3-year follow-up

| Adverse event[a] | *n* (%) |
|---|---|
| Any adverse event | 13 (26.0) |
| Back pain | 5 (10.0) |
| Headache | 4 (8.0) |
| Nausea or vomiting | 3 (6.0) |
| Cough | 1 (2.0) |
| Pyrexia | 1 (2.0) |
| Upper respiratory tract infection | 1 (2.0) |

*SMA* spinal muscular atrophy. [a]Adverse events are counted based on the number of SMA patients received nusinersen treatment

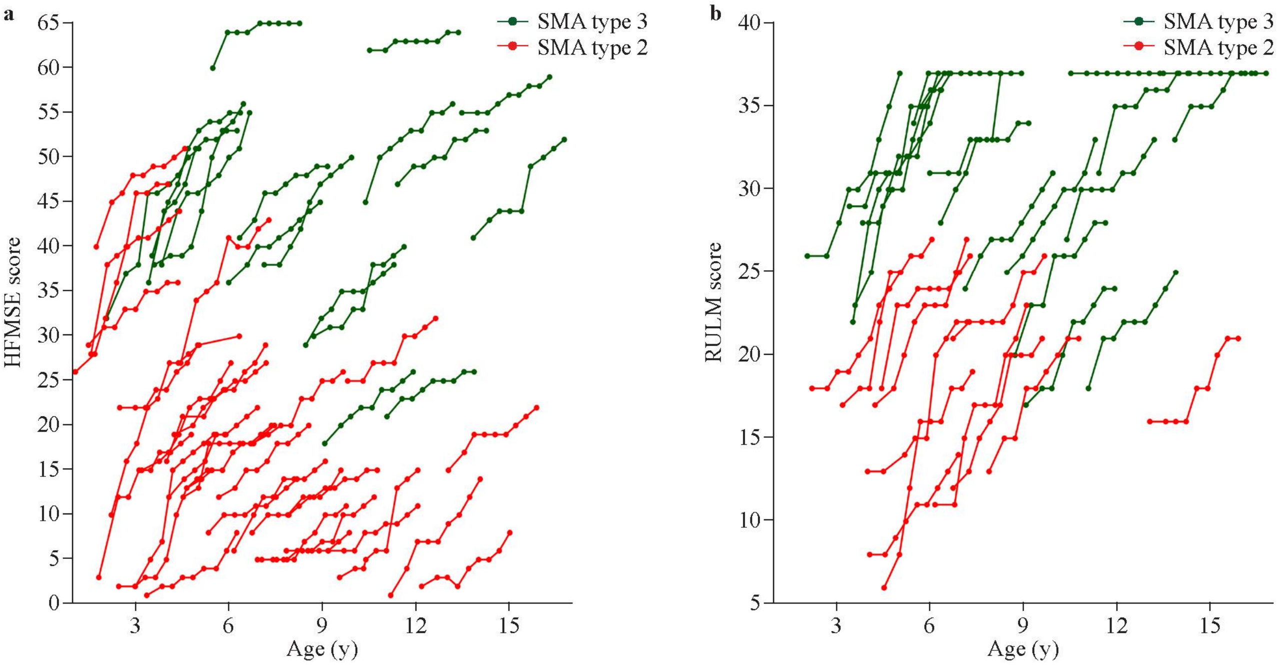


**Supplementary Fig. 1** Evolution of the HFMSE and RULM scores of patients during the follow-up period. Motor function trajectories reflected by the HFMSE and RULM scores in children with SMA types 3 (cyan) and 2 (red). Each line represents one patient. Panels **a** and **b** represent the HFMSE and RULM scores of the study patients, respectively. *HFMSE* Hammersmith Functional Motor Scale Expanded, *RULM* Revised Upper Limb Module, *SMA* spinal muscular atrophy

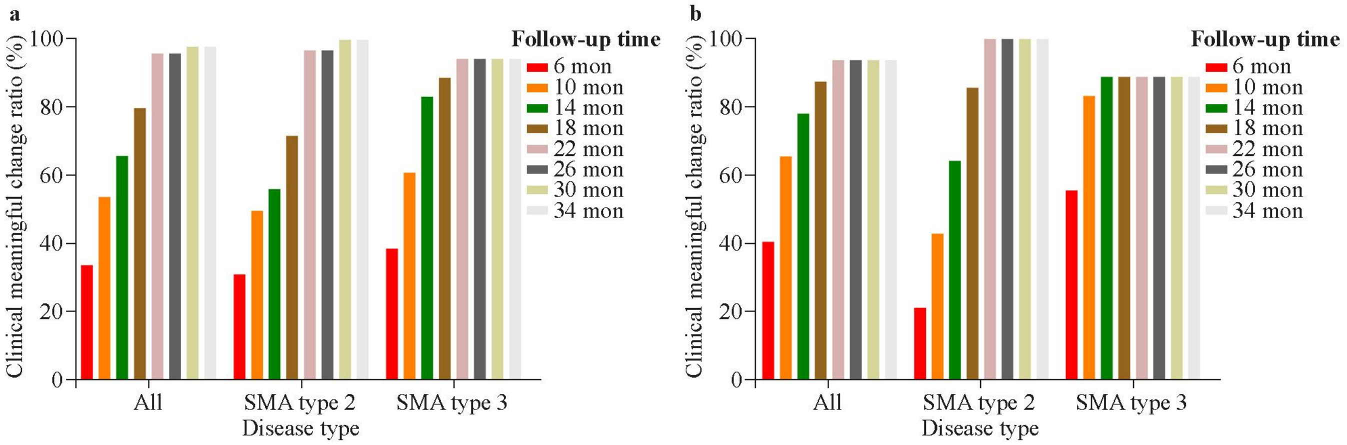


**Supplementary Fig. 2** Proportion of responders to the HFMSE (**a**) and RULM scores (**b**) at each follow-up time point. A change in the HFMSE score of at least 3 points or a change in the RULM score of at least 2 points is considered a clinically meaningful response. *HFMSE* Hammersmith Functional Motor Scale Expanded, *RULM* Revised Upper Limb Module, *SMA* spinal muscular atrophy
